# Whole Genome Sequencing Identifies a Novel Factor Required for Secretory Granule Maturation in *Tetrahymena thermophila*

**DOI:** 10.1534/g3.116.028878

**Published:** 2016-06-09

**Authors:** Cassandra Kontur, Santosh Kumar, Xun Lan, Jonathan K. Pritchard, Aaron P. Turkewitz

**Affiliations:** * Department of Molecular Genetics and Cell Biology, The University of Chicago, Illinois 60637; † Department of Genetics, Stanford University, California 94305; ‡ Howard Hughes Medical Institute, Stanford University, California 94305; § Department of Biology, Stanford University, California 94305

**Keywords:** evolutionary cell biology, endomembranes, secretion, dense core, regulated exocytosis

## Abstract

Unbiased genetic approaches have a unique ability to identify novel genes associated with specific biological pathways. Thanks to next generation sequencing, forward genetic strategies can be expanded to a wider range of model organisms. The formation of secretory granules, called mucocysts, in the ciliate *Tetrahymena thermophila* relies, in part, on ancestral lysosomal sorting machinery, but is also likely to involve novel factors. In prior work, multiple strains with defects in mucocyst biogenesis were generated by nitrosoguanidine mutagenesis, and characterized using genetic and cell biological approaches, but the genetic lesions themselves were unknown. Here, we show that analyzing one such mutant by whole genome sequencing reveals a novel factor in mucocyst formation. Strain UC620 has both morphological and biochemical defects in mucocyst maturation—a process analogous to dense core granule maturation in animals. Illumina sequencing of a pool of UC620 F2 clones identified a missense mutation in a novel gene called *MMA1* (*M*ucocyst *ma*turation). The defects in UC620 were rescued by expression of a wild-type copy of *MMA1*, and disrupting *MMA1* in an otherwise wild-type strain phenocopies UC620. The product of *MMA1*, characterized as a CFP-tagged copy, encodes a large soluble cytosolic protein. A small fraction of Mma1p-CFP is pelletable, which may reflect association with endosomes. The gene has no identifiable homologs except in other *Tetrahymena* species, and therefore represents an evolutionarily recent innovation that is required for granule maturation.

All eukaryotes possess a network of membrane-bound organelles that underlie wide-ranging cellular activities. While the basic features of this network are conserved, there is phylogenetic evidence that specific pathways have tended to experience high levels of innovation over evolutionary time, including pathways directly involved in protein secretion (Kienle *et al.* 2009a, 2009b; Kloepper *et al.* 2007; Dacks and Field 2007). From a cell biological perspective, such innovations are interesting because they may underlie specialized secretory responses. Dense core granules (DCGs) in animal cells are secretory organelles that are adapted for the storage of bioactive peptides (Guest *et al.* 1991; Kelly 1991). These peptides can subsequently be released in response to extracellular stimuli, a phenomenon called regulated exocytosis (Burgoyne and Morgan 1993). Studies of DCG biogenesis, particularly in mammalian endocrine cells, have detailed a biosynthetic pathway in which newly formed DCGs undergo extensive maturation before they are competent for cargo exocytosis (Morvan and Tooze 2008; Steiner 2011). Maturation involves pathway-specific machinery. For example, key proteases—the prohormone convertases—dedicated to generating bioactive peptides, were evolutionarily derived from a *trans*-Golgi network protease called KEX2/furin that is conserved within the Opisthokont lineage (including both fungi and animals) but not demonstrably present in other eukaryotic lineages (Steiner 2011, 1998). More recently, genetic dissection of DCG biogenesis in invertebrates is uncovering numerous additional factors. Some of these are conserved within metazoa, without any clearly homologous genes in other lineages (Ailion *et al.* 2014; Sumakovic *et al.* 2009). Thus, mechanisms underlying the formation of DCGs in animals are likely to depend, in part, on lineage-restricted innovation.

Ciliates are a large clade of unicellular eukaryotes, many of which are striking in their behavioral and structural complexity. This complexity includes a remarkable array of secretory vesicles that contribute to functions including predation, predator deterrence, and encystation (Rosati and Modeo 2003). The ciliate vesicles show marked structural and functional similarities to metazoan DCGs (Turkewitz 2004). However, as members of the SAR (Stramenopile/Alveolate/Rhizaria) lineage, Ciliates are very distantly related to Opisthokonts (Parfrey *et al.* 2006). This immense evolutionary divergence raises the possibility that dense core granules in Ciliates could have arisen largely independently from those in animals (Elde *et al.* 2007; Lukes *et al.* 2009).

Biochemical and other molecular data for ciliate granules are largely limited to two species belonging to the Oligohymenophorean branch: *Tetrahymena thermophila* and *Paramecium tetraurelia* (Briguglio and Turkewitz 2014; Vayssie *et al.* 2000; Lynn and Doerder 2012; Gentekaki *et al.* 2014). The ciliate granule cargo proteins, like many proteins in endocrine granules, are acidic, bind calcium with low affinity, and form large aggregates within the secretory pathway, suggesting that compartment-specific aggregation may be a widespread mechanism for sorting to secretory granules(Chanat *et al.* 1991; Arvan and Castle 1992; Verbsky and Turkewitz 1998; Chilcoat *et al.* 1996; Garreau de Loubresse 1993). Moreover, the ciliate proteins subsequently undergo proteolytic maturation via endo- and exoproteolytic processing, similar to cargo proteins in metazoan endocrine granules (Madeddu *et al.* 1994; Verbsky and Turkewitz 1998). However, neither the ciliate cargo proteins nor the processing enzymes are homologous to their functional counterparts in metazoans, suggesting that unrelated proteins evolved to produce similar functions within the secretory pathway (Kumar *et al.* 2014, 2015; Madeddu *et al.* 1995; Bowman *et al.* 2005b). That idea is also consistent with identification of a set of genes required in *P. tetraurelia* for granule exocytosis, which are lineage-restricted rather than conserved (Bonnemain *et al.* 1992; Gogendeau *et al.* 2005; Froissard *et al.* 2001; Skouri and Cohen 1997). Similarly, while *T. thermophila* and *P. tetraurelia* express large families of classical eukaryotic trafficking determinants such as Rab GTPases and SNAREs, they lack any clear homologs of some specific subtypes that are associated with secretory granules in metazoans (Bright *et al.* 2010; Schilde *et al.* 2010; Bustos *et al.* 2012). Taken together, the current data are consistent with the idea that similar functions in granule formation can be provided in ciliates and animals by paralogs that arose independently within the same gene families, or by unrelated genes.

One potentially powerful approach to expanding our catalog of genes involved in ciliate granulogenesis is forward genetics, which has provided a key tool in dissecting mechanisms of membrane trafficking in budding yeast and other organisms (Novick and Schekman 1980; Horazdovsky *et al.* 1995). Importantly, screens based on random mutagenesis are free of the bias inherent in candidate gene approaches. In *T. thermophila*, mutants with a variety of defects in secretory granule biogenesis have been generated using nitrosoguanidine, and characterized using genetic and cell biological approaches, but none of the underlying genetic lesions has been identified (Orias *et al.* 1983; Melia *et al.* 1998; Bowman *et al.* 2005a; Maihle and Satir 1985). However, advances in high throughput sequencing should make it possible to identify the causative mutations via whole genome comparisons, as has recently been demonstrated for a *T. thermophila* mutant in ciliary basal body orientation (Galati *et al.* 2014).

The secretory granules in *T. thermophila*, called mucocysts, are filled primarily with proteins of the Grl (Granule lattice) family (Cowan *et al.* 2005). The Grl proteins undergo proteolytic processing, which is required for morphological maturation of newly synthesized mucocysts (Verbsky and Turkewitz 1998; Bradshaw *et al.* 2003; Ding *et al.* 1991; Collins and Wilhelm 1981). Mucocysts also contain a second family of abundant proteins(Haddad *et al.* 2002; Bowman *et al.* 2005b). The best-studied member is Grt1p (Granule tip), whose name derives from the polarized distribution of this protein in mature mucocysts (Bowman *et al.* 2005a). Both Grl proprotein processing, and Grt1p polarization, are largely blocked in an exocytosis-deficient mutant, generated by nitrosoguanidine mutagenesis, called UC620 (Bowman *et al.* 2005a). The UC620 mutation showed Mendelian inheritance expected for a recessive allele, and the mucocyst maturation defects were partially suppressed under starvation conditions (Bowman *et al.* 2005a). This suppression is potentially related to the notable upregulation of many genes involved in mucocyst biogenesis under starvation conditions, as judged by transcript abundance (Rahaman *et al.* 2009; Briguglio *et al.* 2013; Kumar *et al.* 2014). We have now used whole genome sequencing, applied to F2 progeny of UC620, to identify the genetic lesion in this mutant. Analysis of the gene, which we call *MMA1* for *M*ucocyst *Ma*turation 1, illustrates the power of forward genetics to uncover ciliate-restricted innovations required for secretory granule biogenesis.

## Materials and Methods

### Cells and cell culture

*T. thermophila* strains relevant to this work are listed in Table 1. Unless otherwise stated, cells were cultured in SPP [2% proteose peptone, 0.2% dextrose, 0.1% yeast extract, 0.003% sequestrene (ferric ethylenediaminetetraacetic acid)] and starved in 10 mM Tris buffer, pH 7.4, both at 30° while shaking at 99 rpm. PP medium (2% proteose peptone, 0.003% sequestrene), for cells grown in 96-well plates, also included 250 μg/ml penicillin G, 250 μg/ml streptomycin sulfate, and 0.25 μg/ml amphotericin B fungizone (Gibco). For most uses, cells were grown overnight to medium density (1.5–3 × 10^5^ cells/ml) in a volume of SPP equal to one-fifth of the nominal culture flask volume. Cell densities were determined using a Z1 Beckman Coulter Counter. Cells in 96-well or drop plates, including cells under drug selection, were grown for 3 d at 30° in moisture chambers. Scoring and screening of cells was done on an inverted microscope at 100× magnification.

**Table 1:** Strains used in the work

Strain	Drug Resistance [Micronuclear Genotype (Macronuclear Phenotype)]	Exocytosis Phenotype	Mating Type	Notes
B2086	*mpr1-1/mpr1-1* (mp-s)	exo^+^	II	Parental
CU428	*mpr1-1/mpr1-1* (mp-s)	exo^+^	VII	Parental (mutagenized)
CU427	*chx1-1/chx-1-1 (cy-s)*	exo^+^	VI	Initial outcross
IA264	*gal1-1/gal1-1 (dg-s)*	exo^+^	II	Outcross, this paper
UC620	*chx1-1/chx1-1;*	exo^-^		
	*mpr1- 1/mpr1-1* (cy-r, mp-r)			
F1	CHX/chx1-1;MPR/mpr1-1; GAL/gal1-1,exo^+^/exo^-^	exo^+^		
	(cy-r, mp-r, dg-r)			
CU428 *MMA1*- CFP	*mpr1-1/mpr1-1* (mp-s); (cy-r)	exo^+^	VII	*MMA1*-CFP::rpL29 cy-R inducibly expressed in CU428
				background
UC620 *MMA1*- CFP	*chx1-1/chx1-1*; *mpr1- 1/mpr1-1* (cy-r, mp-r); (cy- r)	exo^+^		*MMA1*-CFP::rpL29 cy-R inducibly expressed in UC620
				background
∆*mma1*	*mpr1-1/mpr1-1* (mp-s); (pmr-r)	exo^-^	VII	*MMA1* disruption by neo4, in CU428 background

### Genetic crosses

Cultures of strains to be mated were grown to 1.5–3 × 10^5^ cells/ml, washed twice, and resuspended in starvation medium, 10 mM Tris, pH 7.4, to a final density of 2 × 10^5^ cells/ml. Unless otherwise specified, cells were pelleted in 50 ml conical tubes at ∼600–1000 × *g* for 1 min. Aliquots of 10 ml of cells were starved overnight (12–18 hr) at 30° in 100 mm Petri dishes to initiate sexual reactivity. Equal volumes (5 ml) of cells to be mated were then gently mixed, within the 30° incubator, in a new Petri dish. Mating cells were incubated at 30° for 5–8 hr in a moist chamber, and then refed with an equal volume of 2% PP to generate karyonides (Orias and Hamilton 2000; Orias *et al.* 2000; Hamilton and Orias 2000).

### Selecting progen

Beginning 1 hr after refeeding, single mating pairs were isolated by mouth pipetting into individual 30 μl drops of SPP in a Petri dish containing 48 drops in an 8 × 6 grid array. To maximize the number and diversity of progeny established, exconjugants were also isolated by distributing 50 μl/well of mating pairs into a 96-well plate containing 50 µl/well SPP (Orias 2012). Cells were grown, then replicated into separate 96-well plates containing 100 μl/well 2% PP supplemented with the appropriate drug to eliminate drug-sensitive parentals. Selection with cycloheximide (chx) and 6-methyl purine (6-mp) was on 15 μg/ml (both from 1000 × stock; chx stock in 100% methanol), paromomycin (pms) at 100 μg/ml (from 1000 × stock), and 2-deoxygalactose (2-dgal) at 2.5 mg/ml (from 50 × stock)(Orias 2012). After 3 d growth, plates were scored for drug resistance to identify progeny. Cells selected with 2-dgal were scored after 7 d.

### Obtaining drug-sensitive F1 assortants

In outcrosses where one parent contributed an allele conferring drug resistance (chx1-1), exconjugant progeny were initially heterozygous in both the MIC and MAC. Through phenotypic assortment, some subsequent vegetative progeny will lose all copies of the resistance allele in the MAC. We identified these progeny by serial passaging combined with drug sensitivity tests, as described in (Orias 2000).

### Mating type testing

Bacterized medium was prepared by adding *Pseudomonas syringae* to a 25-ml flask of SPP, and growing overnight at 30° with shaking at 225 rpm. This culture was diluted 50-fold in sterile water to constitute 2% bacterized peptone (BP). Clones to be tested were replicated into a 96-well plate with 50 μl/well 2% BP and grown for 3 d. A 100-μl inoculum of each tester strain (MT II, III, IV, V, VI, or VII), taken from an overnight culture, was added to 10 ml 2% BP in Petri dishes and grown for 3 d. Tester strains were obtained from the Tetrahymena Stock Center (https://tetrahymena.vet.cornell.edu/). At that time, 50 μl/well of the tester strains were added separately to each unknown, and plates were incubated at 30°. A control matrix of each tester separately mated to each of the others and itself was also included. Between 4 and 8 hr after mixing, cells were scored for pairing. The mating type was defined for clones that paired with five of the six mating type testers, as equivalent to that of the sole tester strain with which it failed to pair.

### Testing assortants for exocytosis competence using Alcian Blue

Cells were grown in SPP, replicated into a 96-well plate with 50 μl/well 2% BP, and grown for 3 d. Capsule formation was induced by adding 50 μl/well 0.02% Alcian blue in 0.5 mM CaCl_2_ solution to each well, followed by the immediate addition of 25 μl of 2% PP. Wells were scored for the presence of Alcian blue-stained capsules. Clones showing no capsule formation whatsoever were then retested in bulk culture, before being selected for genomic DNA extraction. For this, cells were grown to 3 × 10^5^ cells/ml in 25 ml SPP, washed twice and resuspended in 10 mM Tris, pH 7.4, then starved overnight (12–18 hr) at 30°. Cells were concentrated to 3 ml, and transferred to a new 125-ml flask, stimulated by rapid addition via syringe of 1 ml 0.1% Alcian blue, then diluted after 15 sec with 45 ml 0.25% PP + 0.5 mM CaCl_2_. Cells were washed once in 50 ml Tris, concentrated to 2 ml, and resuspended in 25 ml Tris for screening by microscopy.

### Genomic DNA extraction

Cells were grown to 3 × 10^5^ cells/ml in 25 ml SPP, and starved for 18–24 hr in 10 mM Tris-HCl, pH 7.5; 1.5 ml of cells was then placed in an Eppendorf tube and concentrated by pelleting to 50 μl. Urea buffer (700μl; 42% w/v urea, 0.35 M NaCl, 0.01 M Tris pH 7.4, 0.01 M EDTA, 1% SDS) was added, followed by gentle shaking and subsequent addition of 0.1 mg/ml Proteinase K for a 5-min incubation at 50°. A 750-μl aliquot of phenol:chloroform:isoamyl alcohol (25:24:1) was then added, the tube contents mixed by inversion, and then spun for 15 min at 3500 rpm. The top, aqueous, layers were transferred to new tubes using pipette tips with cut-off ends, and the extraction repeated. The top layers were then extracted with an equal volume of chloroform:isoamyl alcohol (24:1), and mixed with a one-third volume of 5 M NaCl. DNA was precipitated with an equal volume of isopropyl alcohol, gently spooled onto a hooked glass pipette and transferred to a new nonstick Eppendorf tube (Eppendorf LoBind Microcentrifuge tube). DNA was washed and pelleted twice with 1 ml 70% ethanol, and the final pellet left to air dry for 5–10 min. DNA was resuspended in 25 μl 1 × TE, pH 8.0, treated with 2 μl RNAse A (10 mg/ml, Fermentas) overnight at 55°, and stored at –20°.

### Disruption of MMA1 to generate Δmma1 strains

*MMA1* (TTHERM_00566910) was replaced in the Macronucleus with the neo4 drug resistance cassette, generously provided by K. Mochizuki (IMPA, Vienna, Austria) via homologous recombination with the linearized vector pUC620MACKO-neo4 (Supplemental Material, Figure S1). We amplified ∼600 bp of the upstream and downstream flanks of *MMA1* using the primer pairs 034 and 035, and 036 and 037, respectively (Figure S2), and cloned them into the *Sac*I and *Xho*I sites, respectively, of the neo4 cassette using an In-Fusion cloning kit (Clontech, Mountain View, CA). CU428 cells were then biolistically transformed with the final construct pUC620MACKO-neo4, linearized with *Not*I and *Sal*I (Chilcoat *et al.* 1996; Kumar *et al.* 2014). Transformants were selected on the basis of paromomycin resistance, then serially transferred for 3–4 wk in increasing drug concentrations to drive fixation of the null allele.

### RT-PCR confirmation of MMA1 disruption

Cultures were grown to 1.5–3.0 × 10^5^ cells/ml, washed, and starved for 2 hr in 10 mM Tris pH 7.4. Total RNA was isolated as per manufacturer’s instructions using RNeasy Mini Kit (Qiagen, Valencia, CA). The presence of *MMA1* transcripts was assayed with the OneStep RT-PCR kit (Qiagen) using primers (083 and 073, Figure S2) to amplify ∼650 bp of the *MMA1* gene. Gene knockout was confirmed by the continued absence of the corresponding transcripts after ∼2 wk of growth in the absence of drug selection (four to five serial transfers/wk). To confirm that equal amounts of cDNA were being amplified, control RT-PCR with primers specific for the α-tubulin gene were run in parallel.

### Vector construction and expression of the MMA1-CFP gene fusion

The *MMA1* gene was cloned into the pBSICC Gateway vector (a gift from D. Chalker, Washington University, St. Louis, MO), using the primers listed in Figure S2. Briefly, *MMA1*, minus the stop codon, was PCR-amplified using primers 091 and 092, and recombined into a destination vector (pIBCC) containing an *MTT1*-inducible CFP tagged expression cassette cloned upstream of a cycloheximide-resistant rpl29 allele. For transformation, the construct was linearized with BaeI and *Spe*I, and biolistically transformed into the wild type CU428 or mutant UC620 cell line (Kumar *et al.* 2014).

### Biolistic transformation

*Tetrahymena* were transformed by biolistic transformation as previously described (Kumar *et al.* 2014; Chilcoat *et al.* 1996). Afterward, filters were transferred to a flask with 50 ml prewarmed (30°) SPP without drug, and incubated at 30° with shaking for 4 hr. Transformants were then selected by adding paromomycin (120 μg/ml with 1 μg/ml CdCl_2_), or cycloheximide (12 μg/ml). Cells were scored for drug resistance after 3 d (paromomycin) or 5 d (cycloheximide). Transformants were serially transferred 5 d a week in decreasing concentrations of CdCl_2_ and increasing concentrations of drug. In all cases, selection was for at least 2 wk before further testing (Chalker 2012).

### Immunofluorescence

To visualize mucocysts, cells were fixed, permeabilized with detergent, immunolabeled with mAb 5E9 (10%) or mAb 4D11 (20%) hybridoma supernatant, and analyzed as described previously (Bowman and Turkewitz 2001; Kumar *et al.* 2014). After a 2-hr induction by 2 µg/ml CdCl_2_, Mma1p-CFP fusion protein was visualized using Rabbit anti GFP (Invitrogen) (1:400), followed by Alexa 488-conjugated anti-Rabbit antibody (1:250). For simultaneous imaging of Mma1p-CFP and Grl3p, cells were costained using the mAb 5E9 as previously described, and with the polyclonal anti-GFP antibody (Life Technologies) diluted 1:400 in 1% BSA. Cells were then coincubated with the 2° antibodies Texas red-coupled goat anti-mouse IgG diluted 1:99, and 488-coupled donkey anti-rabbit IgG diluted 1:250 in 1% BSA. Cells were imaged using a Leica SP5 II Confocal Microscope, and image data were analyzed as previously described (Kumar *et al.* 2014). Images were captured with the LAS_AF confocal software (Leica) for Windows 7. Image data were colored and adjusted for brightness/contrast using ImageJ.

### Dibucaine stimulation

Dibucaine stimulation of exocytosis was performed as described previously (Rahaman *et al.* 2009).

### Subcellular fractionation

Cells were grown to 3 × 10^5^/ml, and then transferred into 10 mM Tris, pH 7.4. Transgene expression was induced using 0.25 µg/ml CdCl_2_ for 2 hr at 30°. Cells were chilled and centrifuged (1000 × *g*) in a clinical centrifuge for 1 min. All subsequent steps were at 4°. Cells were resuspended and washed once in Buffer A (20 mM HEPES-KOH pH 7.0, 38 mM KCl, 2 mM MgCl_2_, and 2 mM EGTA) and the pellet volume measured. The pellet was resuspended in three volumes of Buffer B (20 mM HEPES-KOH pH 7.0, 38 mM KCl, 2 mM MgCl_2_, 2 mM EGTA, and 0.3 M sucrose) containing protease inhibitor cocktail tablet (Roche). Cells were passed through a ball-bearing cell cracker with nominal clearance of 0.0004 inches. The homogenate was centrifuged for 30 min at 10,000 × *g*. To separate cytosolic and membrane fractions, that supernatant was further centrifuged for 1 hr at 100,000 × *g*. After centrifugation, supernatant (cytosolic) and pellet (membrane) fractions were dissolved in SDS-PAGE buffer and incubated for 15 min at 90°.

### Tricholoracetic acid precipitation of whole cell lysates

Cells (∼3 × 10^5^)cells were pelleted, washed twice with 10 mM Tris pH 7.4, and precipitated with 10% trichloroacetic acid (TCA). Precipitates were incubated on ice for 30 min, centrifuged (18,000 × *g*, 10 min, 4°), washed with ice-cold acetone, repelleted (18,000 × *g*, 5 min, 4°) and then dissolved in 2.5× SDS-PAGE sample buffer.

### Immunoprecipitation

CFP-tagged fusion protein was immunoprecipitated (for western blot) from detergent lysates using polyclonal rabbit anti-GFP antiserum as described previously (Briguglio *et al.* 2013).

### Western blotting

Samples were resolved by SDS-PAGE and transferred to 0.45 μm PVDF membranes (Thermo Scientific, Rockford, IL). Blots were blocked and probed as previously described (Turkewitz *et al.* 1991). The rabbit anti-Grl1p, rabbit anti-Grl3p, rabbit anti polyG (Xie *et al.* 2007), and mouse monoclonal anti-GFP (Covance, Princeton, NJ) 1° antibodies were diluted 1:2000, 1:800, 1:10,000, and 1:5000, respectively. Protein was visualized with either ECL Horseradish Peroxidase-linked anti-rabbit (NA934), or anti-mouse (NA931) (Amersham Biosciences, Buckinghamshire, England) 2° antibody diluted 1:20,000, and SuperSignal West Femto Maximum Sensitivity Substrate (Thermo Scientific, Rockford, IL).

### Gene expression profiles

Expression profiles were derived from the *Tetrahymena* Functional Genomics Database (http://tfgd.ihb.ac.cn/), with each profile normalized to that gene’s maximum expression level (Xiong *et al.* 2013; Miao *et al.* 2009).

### In silico analyses

Alignment of protein sequences was performed using CLUSTALX (1.8) with default parameters.

### Library construction

DNA was sonicated to produce a 300–400 bp distribution, and then end-repaired and A-tailed enzymatically using Illumina’s standard TruSeq DNA library preparation. These products were ligated to Illumina Truseq adapters. Libraries, following indexing via PCR, were size selected to remove adapter and PCR dimers, pooled, and cosequenced on two lanes of the Illumina HiSequation 2500 using a 2 × 100 base pair format. The unique sequences incorporated during library construction were subsequently used to identify the source of each read, using postsequencing bioinformatics.

### Whole genome sequencing

Sequencing of the macronuclear DNA library was performed on an Illumina HiSequation 2500 by the University of Chicago Genomics Facility at the Knapp Center for Biomedical Discovery (KCBD). The sequencing process followed the manufacturer’s instructions, and the sequence files (fastq) were produced using the Illumina demultiplexing software CASAVA (v1.8). A total number of 269 million paired-end reads (2 × 101 bp) were generated. The F2 lines with the mutation of interest were sequenced to ∼70-fold genome coverage. The parental strain, *i.e.*, the wildtype background upon which the mutations were initially induced, was sequenced to ∼256-fold genome coverage. The two strains that were used in outcrossses to generate the F2 lines were sequenced to ∼65- and ∼73-fold genome coverage, respectively. Genome sequencing of *Tetrahymena* primarily reflects the Macronuclear genome, in which genes are generally present at ∼45 copies compared to the two copies in the Micronucleus. Because the Micronucleus represents the germline nucleus, only Micronuclear alleles are transmitted to progeny (Karrer 2000). However, because all parental lines are wild type regarding exocytosis except for UC620 itself, we made the simplifying assumption that bulk DNA sequencing of mutant *vs.* parental lines would permit us to identify the causative mutation in UC620.

### Sequence alignment

Reads from total genomic DNA sequencing were mapped to the *T. thermophila* Macronuclear genome sequence released by Broad institute using the Burrows-Wheeler Aligner software (BWA) version 0.5.9 (Eisen *et al.* 2006; Coyne *et al.* 2008; Li and Durbin 2009). Default parameters were used when running bwa aln except the following: 1) “Maximum edit distance” was set to “2”; 2) “Maximum number of gap opens” was set to “0”; 3) “Number of threads” was set to “8”; and 4) “Iterative search” was disabled. The output files were converted to bam files using the “sampe” utility of the BWA software with default parameters, and the SAMtools software version 0.1.18 (Li *et al.* 2009). SAMtools was then used to sort the bam files and remove PCR duplicates. Picard Tools version 1.92 was used to add group names to the bam files with parameter “VALIDATION_STRINGENCY” set to “LENIENT” (http://picard.sourceforge.net). The resulting bam files were indexed by SAMtools.

### Variants discovery

The Genome Analysis Toolkit (GATK) version 2.5-2 was applied to identify variants with total genomic DNA sequencing data of the five samples (McKenna *et al.* 2010). The following steps were taken in this procedure. 1) Realignment of reads using RealignerTargetCreator and IndelRealigner tool of the GATK package to correct the misalignment caused by site mutations, insertions and deletions; and 2) variants were called using the HaplotypeCaller tool of the GATK package with the “out_mode” set to “EMIT_ALL_CONFIDENT_SITES” that produces calls at variant sites and confident reference sites; “stand_call_conf” set to “50.0” and “stand_emit_conf” set to “30.0”. Full documentation of parameters can be found at https://www.broadinstitute.org/gatk/guide/tooldocs/org_broadinstitute_gatk_tools_walkers_haplotypecaller_HaplotypeCaller.php#-output_mode. A total number of 67,256 variants was called with this procedure. Among these, 23,493 were single-nucleotide variants (SNVs), which resulted in a SNV density of ∼0.23 kbp^–1^ for a genome of a mappable size of 103,014,375 bp (Coyne *et al.* 2008).

### Candidate screening

The candidate causal variants of the cell exocytosis dysfunction phenotype were selected with the following steps. 1) Variants with missing values for the genotypes were filtered, *i.e.*, a variant will be filtered out if GATK is unsure of the genotype of the variant in any of the samples. 2) Candidate sites had to be homozygous in all strains, and the mutant F2 strain genotype must be different from the genotype of the parental strain and wild-type strains. A total number of 28 such candidate sites was found. Among these 28 variants, 10 were on chromosome 1, seven were on chromosome 2, four were on chromosome 3, one was on chromosome 4, four were on chromosome 5, and two were undefined.

### Data availability

Data and reagents from this work will be made freely available upon request for noncommercial purposes.

## Results

### Generation of F2 clones for sequencing

The concentration of nitrosoguanidine used for mutagenesis to generate UC620 is expected to produce a large number of mutations in each cell (Bowman *et al.* 2005a). For that reason, we could not usefully compare the genomes of UC620 to that of the unmutagenized parent strain, as this would be confounded by the large background of SNVs unrelated to the mutation of interest. To partially overcome this problem, we outcrossed UC620 with strains bearing useful drug resistance markers, and used drug resistance to select progeny that would be heterozygous in the germline Micronuclei for the UC620 mutation. We then mated several progeny clones with one another, and derived the F2 progeny from isolated mating pairs. Since the UC620 mutation is recessive, 1/4 of the progeny, namely those homozygous for the mutant alleles, should display the UC620 phenotype. We therefore focused on matings producing the expected 1:3 ratio of mutant:wildtype exocytosis phenotypes among the progeny. A flowchart of the overall genetic strategy is shown in Figure S3.

Identifying the homozygous mutant F2 progeny was complicated by the phenomenon of phenotypic assortment, which derives from the amitotic division of the Macronucleus during cytokinesis (Karrer 2000). In particular, even within clones of cells heterozygous for the UC620 mutation, some cells will lose most, or all, wildtype alleles in the Macronucleus, due to the random assortment of Macronuclear alleles at each cell division (Doerder *et al.* 1992). For this reason, we judged the homozygous mutant clones to be those showing no wild-type phenotypes whatsoever, in tests performed initially in 96-well plates, and confirmed in bulk cultures. We obtained 25 such clones, derived from two matings.

DNA was prepared from the individual clones, and then pooled for sequencing. In parallel, we prepared and sequenced DNA from the strains used during the initial mutagenesis to create UC620, as well as from the strains used for the outcrosses described above.

### A tetrahymenid-restricted gene on chromosome 1 represents a candidate for the mutated gene in UC620

**Figure 1:**
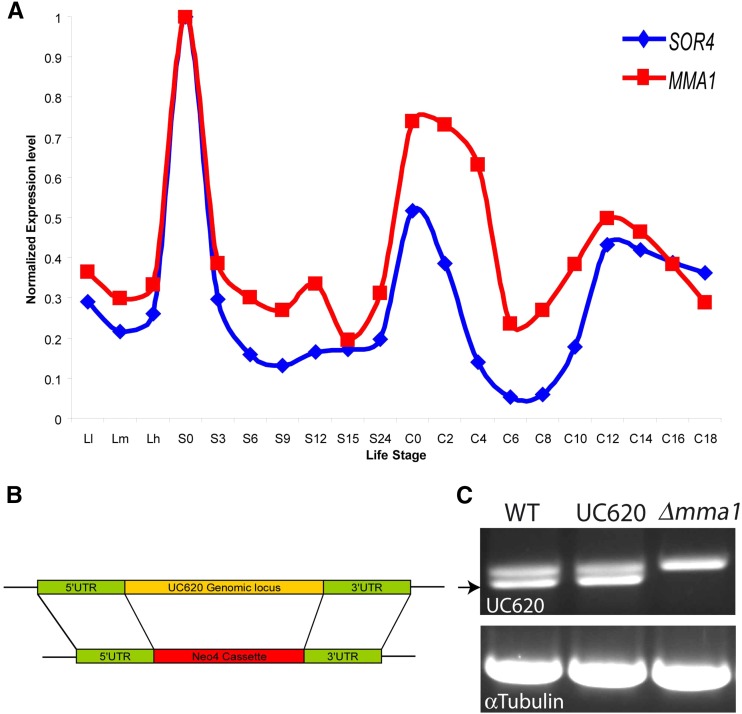
Expression profiling and genomic knockout of *MMA1*. (A) The expression profile of *MMA1* (red trace) is highly similar to that of *SOR4* (blue trace), which encodes a receptor required for mucocyst biogenesis. The profiles of transcript abundance under a variety of culture conditions, derived via hybridization of stage-specific cDNAs to whole genome microarrays, are from the Tetrahymena Functional Genomics Database (http://tfgd.ihb.ac.cn/). In the plots shown, each trace was normalized to that gene’s maximum expression level. The culture conditions sampled at successive time points represent growing (L-l, L-m, and L-h), starved (S-0, S-3, S-6, S-9, S-12, S-15, and S-24), and conjugating (C-0, C-2, C-4, C-6, C-8, C-10, C-12, C-14, C-16, and C-18) cultures. For details on the sampling conditions, see Miao *et al.* (2009). (B) Schematic of *MMA1* Macronuclear gene knockout construct. Replacement of the Macronuclear *MMA1* gene by the Neo4 drug resistance cassette was targeted by homologous recombination. (C) Confirmation of *MMA1* Macronuclear knockout by RT-PCR. RNA was extracted from wild type, UC620, and ∆*mma1*, converted to cDNA, and PCR amplified using *MMA1*-specific primers listed in Table S1. A 1% ethidium bromide-stained agarose gel is shown. The *MMA1* product (indicated by arrow), which was confirmed by sequencing, was present in wild type and UC620 samples but absent in ∆*mma1*. The larger amplicon present in all lanes corresponds to *MMA1* with the intron still present, as determined by sequencing, and thus likely reflects amplification of a genomic (Micronuclear) DNA contaminant. Parallel amplification of the α−tubulin gene from all samples was used to control for sample loading.

A total of 28 SNVs was identified that were homozygous in the mutant pool but not present in the parental strains. The criteria used in SNV discovery are detailed in *Materials and Methods*. Of the 28, 10 fell on chromosome 1, to which the UC620 mutation had previously been mapped (Bowman *et al.* 2005a). Four of these chromosome 1 candidates fell within previously annotated genes. Among these, we focused on TTHERM 00566910 based on its expression profile. An online database of *T. thermophila* gene expression allows one to visualize the expression profiles of all genes in this organism, over a range of culture conditions (Xiong *et al.* 2013). Using this database, we previously discerned that a large number of genes associated with mucocyst biogenesis had nearly identical expression profiles (Rahaman *et al.* 2009; Kumar *et al.* 2014). TTHERM 00566910, which we have called *MMA1* for *M*ucocyst *ma*turation, is expressed at under twice the corrected background for the whole genome dataset (Miao *et al.* 2009). However, the shape of the expression profile was strikingly similar to that of known mucocyst-associated genes (Figure 1A). This profile was not shared by other genes harboring SNVs on chromosome 1 (unpublished data). On this basis, *MMA1* emerged as the prime candidate for the gene underlying the defect in UC620.

*MMA1* has clear homologs in several recently sequenced Tetrahymena species (*T. malaccensis*, *T. ellioti*, and *T. borealis*) (Figure S4). Alignment of these genes, together with extensive RNAseq data from *T. thermophila*, facilitated confident assignment of exon–intron boundaries, as well as start and stop codons, for this previously undescribed gene. Based on this annotation, the SNV identified in UC620 changed the *MMA1* stop codon (TGA) to TGT, which encodes cysteine. The mutation therefore potentially results in an aberrant polypeptide product containing an additional 180 amino acids. Except for the homologs in these other Tetrahymenids, we could not identify *MMA1* homologs in any other species.

### Disruption of MMA1 blocks synthesis of docked mucocysts

To ask whether a defect in *MMA1* might account for the mutant phenotype in UC620, we disrupted *MMA1* in wildtype cells by homologous recombination with a drug resistance cassette (Figure 1B). The resulting ∆*mma1* cells had no detectible *MMA1* transcript (Figure 1C). They grew at wild-type rates, indicating that the gene is not essential for normal growth under laboratory conditions.

**Figure 2:**
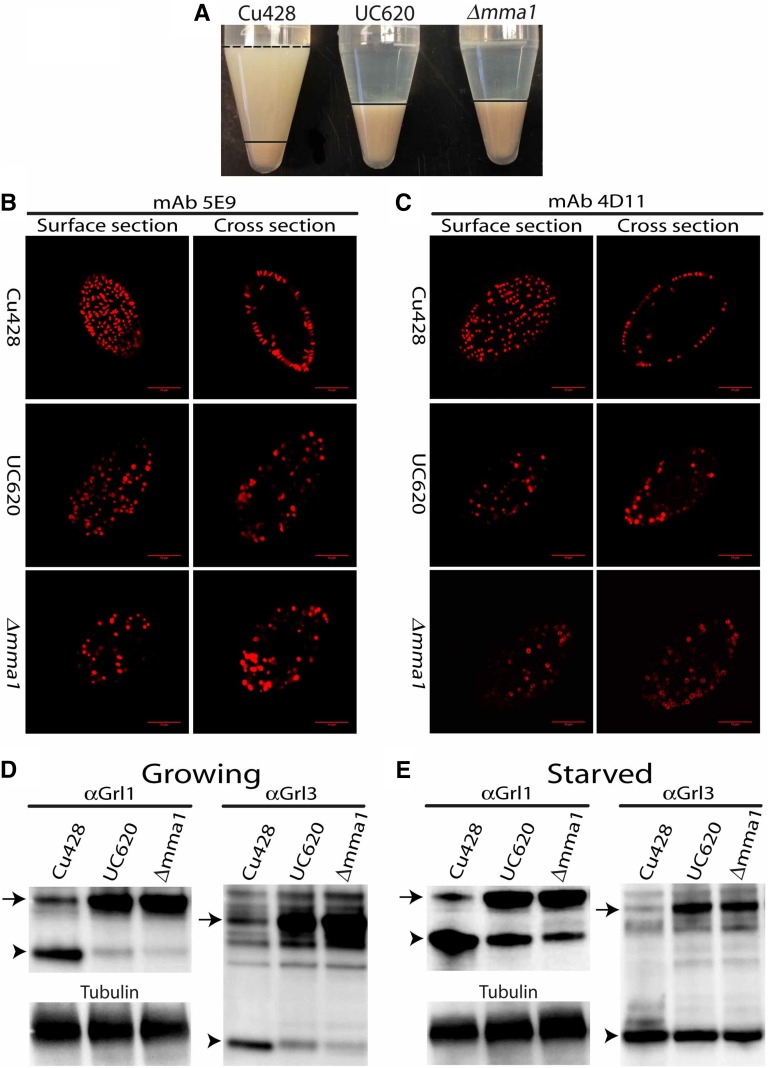
*MMA1* knockout produces a phenocopy of the UC620 mutation. (A) Equal numbers of CU428 wildtype, UC620 and ∆*mma1* cells were stimulated with dibucaine, which promotes global mucocyst exocytosis, and then centrifuged. In wildtype cells, this results in a pellet of cells with an overlying flocculent consisting of the released mucocyst contents. For clarity, the flocculent layer is delineated with a dashed line at the upper border, and an unbroken line at the lower border. In contrast, neither UC620 nor ∆*mma1* produces detectible flocculent. The pellet in wildtype (layer beneath solid line) is smaller than in the mutants because many wild-type cells are trapped in the flocculent. (B and C) Wild-type, UC620, and ∆*mma1* were fixed, permeabilized, and stained with antibodies against two mucocyst core proteins, Grl3p (left panels, 5E9 antibody), and Grt1p (right panels, 4D11 antibody). Shown are optical sections of individual cells, at the cell surface, and a cross section. Wildtype CU428 accumulate docked mucocysts, visible as elongated vesicles that are highly concentrated at the cell periphery. UC620 and ∆*mma1*, in contrast, do not form elongated vesicles, and Grl3p and Grt1p are found in vesicles throughout the cells. The Grt1p signals in both UC620 and ∆*mma1* often appear localized to the vesicle perimeter, while in wildtype cells the signal is localized to the docked mucocyst tips. The scale bars represent 10 μm. (D and E) Whole cell lysates of growing (D), or 6-hr starved (E) cells were resolved by 4–20% SDS-PAGE, and transfers were immunoblotted with antibodies against Grl1p or Grl3p. The unprocessed (pro-Grl) and processed forms of the Grl proteins are indicated by arrows and arrowheads, respectively. In CU428, Grl proteins accumulate primarily in the fully processed form. In contrast, the pro-Grl forms predominate in growing cultures of UC620 and ∆*mma1*. For both UC620 and ∆*mma1*, the defect in processing proGrl proteins is partially rescued under starvation conditions (E). To demonstrate equivalent loading, all samples were immunoblotted in parallel with anti-tubulin antibody (D and E). Each lane in (D) and € represents 10^3^ cell equivalents.

Strikingly, the ∆*mma1* cells demonstrated a strong defect in mucocyst exocytosis. The complete failure to release mucocyst contents upon cell stimulation was identical to that of UC620, when tested with the secretagogues Alcian blue (not shown) or dibucaine (Figure 2A). This defect was due to the failure of ∆*mma1* cells to synthesize mature docked mucocysts, as revealed by indirect immunofluorescence using antibodies against two mucocyst cargo proteins, Grl3p and Grt1p. The ∆*mma1* cells in growing cultures did not accumulate Grl3p in docked mucocysts (Figure 2B). Instead, as in UC620, the Grl3p localized to relatively homogeneous cytoplasmic vesicles.

Similarly, a 2nd mucocyst protein, Grt1p, was mislocalized in both UC620 and ∆*mma1* cells. In wildtype cells, Grt1p resides in docked mucocysts (Figure 2C). In both UC620 and ∆*mma1*, Grt1p instead localizes in cytoplasmic vesicles, frequently appearing as a ring around the vesicle periphery (Figure 2C).

### Δmma1 cells are defective in proteolytic maturation of mucocyst contents

In UC620, the mislocalization of Grl3p and other Grl-family proteins is accompanied by their aberrant biochemical maturation: the Grl pro-protein precursors largely fail to undergo the proteolytic processing that occurs during mucocyst maturation in wild-type cells (Bowman *et al.* 2005a). The ∆*mma1* cells showed the same defect in pro-Grl processing, as shown for two different Grl proteins (Figure 2D). Moreover, the processing defect in ∆*mma1* was partially suppressed when cells were transferred to starvation conditions, as we had previously reported for UC620 (Figure 2E) (Bowman *et al.* 2005a). These results are consistent with the idea that *MMA1* represents the affected gene in the UC620 mutant.

### Mma1p appears to be cytosolic and partially membrane-associated

**Figure 3:**
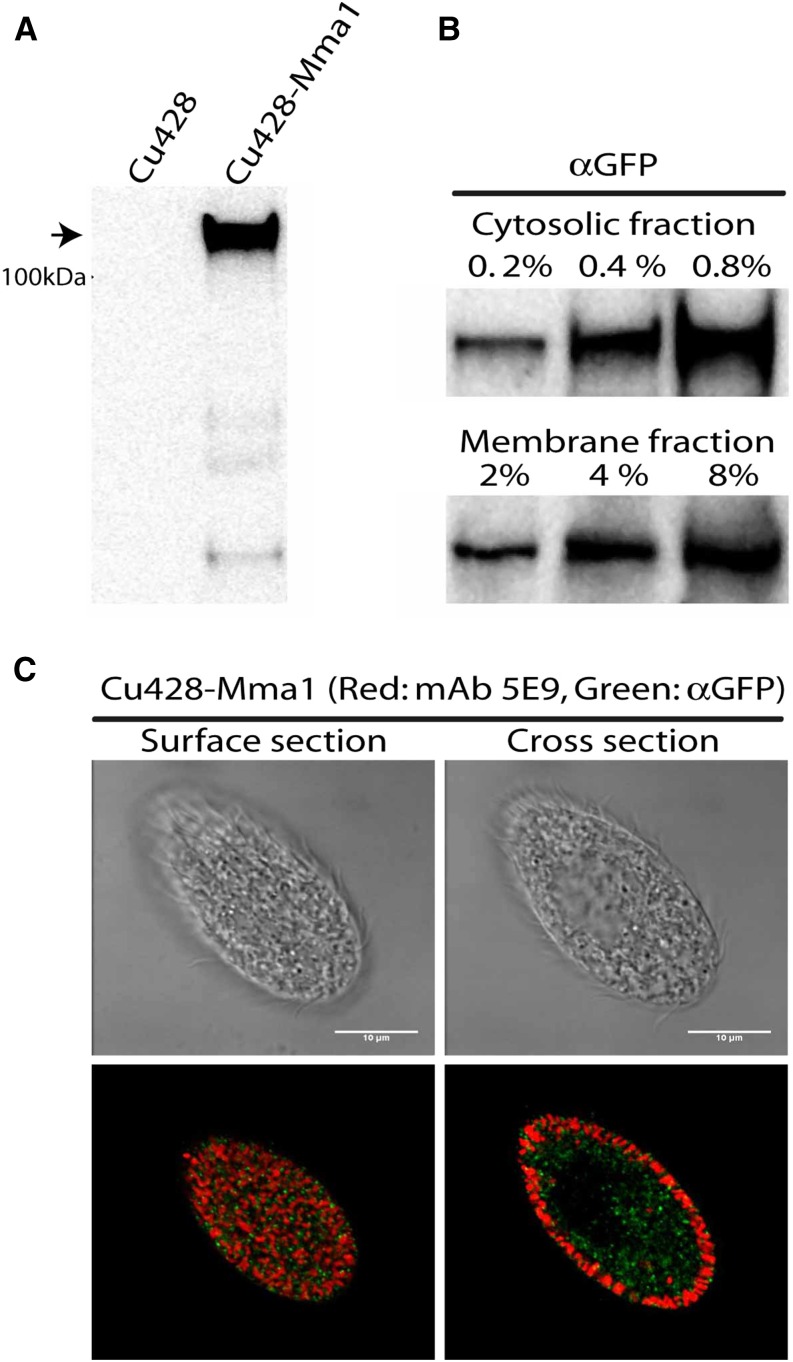
Expression and characterization of Mma1p-CFP. (A) Expression of *MMA1*-CFP. Mma1-CFP fusion protein was immunoprecipitated from detergent cell lysates using anti-GFP antiserum, with wildtype CU428 cells processed in parallel. Immunoprecipitates were subjected to 4–20% SDS-PAGE, and PVDF transfers blotted with anti-GFP mAb. An immunoreactive band (arrowhead) of the size expected for the Mma1p-CFP fusion is seen only in cells expressing this construct. (B) Cells expressing Mma1p-CFP were grown to 3 × 10^5^/ml, and then fractionated into soluble (cytosolic) and pelletable (membrane) fractions, as described in *Materials and Methods*. Samples were separated by SDS-PAGE and immunoblotted with anti-GFP mAb. Three different loadings are shown for each sample, corresponding to 0.2–0.8% of the entire cytosolic fraction, and 2–8% (*i.e.*, 10 × higher cell equivalents) of the membrane fraction; ∼10% of the Mma1p-CFP is found in the membrane fraction. (C) After induction as in (A), cells were fixed, permeabilized, and immunolabeled with rabbit anti-GFP and mAb 5E9, followed by 2° fluorophore-coupled Abs. The scale bars represent 10 μm. Mma1p-CFP (green) appears in small puncta through the cytoplasm, with no significant overlap with docked mucocysts (red). Wildtype cells processed in parallel showed no significant labeling in the green channel (not shown).

A hydropathy plot based on the primary sequence of Mma1p did not reveal either a signal sequence, as would be expected for a protein translocated into the secretory pathway, nor any hydrophobic stretches that could function as transmembrane helices (not shown). Thus the protein is likely to be cytosolic. We expressed a CFP-tagged copy of *MMA1* under the control of the strong and inducible *MTT1* (metallothionein 1) promoter (Shang *et al.* 2002). We resorted to this overexpression strategy because the very low level expression of wildtype *MMA1* made it impossible to detect the expression of 3 × GFP-tagged *MMA1* expressed at the endogenous locus (unpublished data). Mma1p-CFP, immunoprecipitated from whole cell lysates, appeared by Western blotting as a band of the expected size (Figure 3A). We fractionated cells expressing Mma1p-CFP by cracking them with a ball bearing homogenizer, and then subjecting the cleared lysate to high-speed (100,000 × *g*) centrifugation. The protein was found primarily in the soluble fraction, but roughly 10% was found in the high-speed pellet (Figure 3B). Visualization of the CFP in these cells by indirect immunofluorescence showed a large number of small puncta present throughout the cell, but no significant colocalization with mature docked mucocysts (Figure 3C). Taken together, these results suggest that Mma1p-CFP is a cytosolic protein that is partially associated with small vesicles.

### Expression of Mma1p-CFP rescues the UC620 mutant

**Figure 4:**
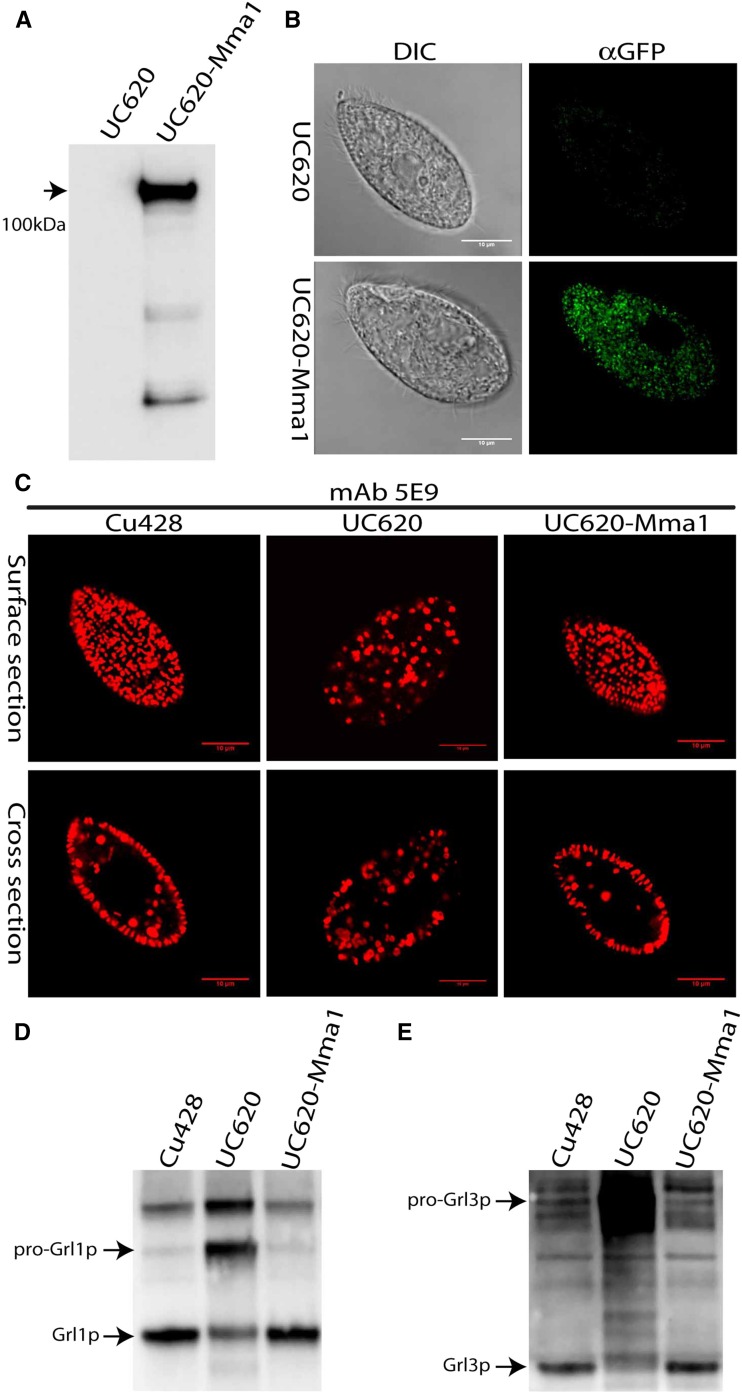
Expression of *MMA1*-CFP rescues the UC620 mutant. (A) Detergent lysates of UC620, or UC620 expressing *MMA1*-CFP, were immunoprecipitated with anti-GFP Ab, and the precipitates separated by SDS-PAGE and immunoblotted with anti-GFP mAb. Cells expressing *MMA1*-CFP show a band of the expected size for the fusion protein (arrow). (B and C) After induction of *MMA1*-CFP with CdCl_2_, cells were fixed, permeabilized, and immunolabeled with rabbit anti-GFP (B) or mAb 5E9 (C), followed by 2° fluorophore-coupled Abs. The scale bars represent 10 μm. (B) Mma1p-CFP localizes to multiple cytoplasmic puncta. (C) The expression of the fusion protein in UC620 cells restores the wild-type pattern of docked mucocysts. (D and E) *MMA1*-CFP expression was induced for 2 hr in growing cell cultures, and wild type and UC620 cultures were in parallel treated with CdCl_2_. Whole cell lysates were resolved by 4–20% SDS-PAGE, and transfers were immunoblotted with antibodies against Grl1p (D) or Grl3p (E). The expression of *MMA1*-CFP in UC620 rescues the processing defect seen for both Grl proteins in UC620. Each lane represents 10^3^ cell equivalents.

To ask whether the CFP-tagged copy of Mma1p was active, we introduced the identical construct into UC620 cells. Since the mutation in UC620 behaves as a recessive allele, the defects in these cells should be rescued by expression of a wild-type copy of the affected gene. A polypeptide of the expected size for the fusion protein was detected by Western blotting, and was localized to a large number of small cytoplasmic puncta (Figure 4, A and B). Importantly, UC620 cells expressing *MMA1*-CFP were indistinguishable from wild type in both mucocyst accumulation, and in pro-Grl processing (Figure 4, C, D, and E). The full rescue of UC620 cells by expression of *MMA1* provides confirmatory evidence that whole genome sequencing has allowed us to identify the genetic lesion in UC620, and that *MMA1* represents a novel factor required for mucocyst maturation.

## Discussion

*T. thermophila* possesses many characteristics that make it an attractive model organism for investigating cell biological questions (Collins and Gorovsky 2005). One particularly promising approach to dissect a variety of pathways has been forward genetics following chemical mutagenesis. The cells are diploid, but can be rapidly brought to homozygosity via a specialized mating, thereby facilitating screening for recessive mutations (Cole and Bruns 1992). More generally, starved cultures of compatible mating types will undergo highly synchronous mating, and the cells are large enough to be readily isolated as individual pairs, which facilitates classical genetic analysis (Orias 2000).

Using these and related tools, > 20 secretion mutants were previously generated by nitrosoguanidine mutagenesis, and analyzed to various degrees (Orias *et al.* 1983; Melia *et al.* 1998; Bowman *et al.* 2005a). All of them fail to release mucocyst contents upon stimulation, and the underlying cell biological defects range from a block in mucocyst synthesis to defects in exocytosis *per se*. However, none of the genetic lesions has ever been identified, thus limiting the insights gained from this collection. Similarly, the literature contains detailed analysis of *Tetrahymena* mutants with defects in features such as cortical pattern formation, cell size, lysosomal enzyme release, cell surface antigen expression, food vacuole formation, motility, and rDNA maturation (Frankel 2008; Hunseler *et al.* 1987; Basmussen and Orias 1975; Pennock *et al.* 1988; Kapler *et al.* 1994; Doerder *et al.* 1985), but in only a single recent case has the gene responsible been identified (Galati *et al.* 2014). That work, by Pearson and colleagues, used a whole genome sequencing approach similar to that which we independently pursued and report in this paper. Our results therefore suggest that mutagenesis linked with whole genome sequencing should be considered a highly accessible approach to link defined pathways with their underlying genes in this organism. This is relevant both for revisiting previously characterized mutants, as well as for developing new genetic screens. Recently, next generation sequencing applied to the ciliate *P. tetraurelia* has illuminated a set of mutants first characterized many decades ago (Singh *et al.* 2014).

Our sequencing strategy involved first outcrossing UC620 to generate heterozygous F1 clones, and then using an F1 × F1 cross to regenerate homozygous mutant progeny. In principle, the second step could have been simplified by crossing the F1 lines with a so-called star (*) strain, employing a strategy called uniparental cytogamy (Cole and Bruns 1992). Conjugation in *Tetrahymena* involves the reciprocal exchange of haploid meiotic products between the pairing cells. A cell that conjugates with a * cell fails to receive a viable haploid pronucleus, and this promotes the endoreduplication of its own haploid genome. As a result, such * crosses can produce whole-cell homozygotes, which would be ideal for screening and sequencing (Cole and Bruns 1992). Star crosses are also known to produce additional, poorly characterized, outcomes at low frequency, but this has not detracted from their usefulness for many applications. However, in early experiments we found that a significant fraction of the progeny of the uniparental cytogamy crosses, isolated as individual pairs, showed drug-resistance phenotypes inconsistent with simple endoreduplication, and we therefore employed the somewhat more laborious F1 × F1 strategy.

One key element in quickly identifying the causative SNV in UC620 was our prior physical mapping. Such mapping is relatively straightforward in *Tetrahymena* due to the availability of a panel of so-called ‘nullisomic’ strains, each bearing a homozygous deletion of an entire micronuclear chromosome, and available from the Tetrahymena Stock Center (Bruns and Brussard 1981). By crossing a strain bearing a recessive mutation with the panel of nullisomics, one can quickly identify the micronuclear chromosome on which the mutation resides. By this approach, the UC620 mutation was mapped to chromosome 1 (Bowman *et al.* 2005a). By using an additional panel of available strains with partial chromosomal deletions, this approach may be extended to map mutations to chromosome arms. However, we failed to obtain viable progeny using some of the partial deletion strains on chromosome 1. In future, it might be very valuable to generate a larger panel of partial chromosomal deletions to cover the *Tetrahymena* genome. If a fine scale panel were available, the mapping of a mutation might be sufficient to then directly identify a small number of candidate genes by sequencing. This approach would bypass the multiple crosses, mating type testing, and progeny screening that we employed to identify *MMA1*.

The mutation identified in the UC620 strain was predicted to change the *MMA1* stop codon, thereby potentially producing a longer polypeptide product. We found unambiguous *MMA1* homologs in the recently sequenced genomes of three other Tetrahymena species, and these alignments helped to confirm the stop codon assignment. The stop codon mutation may result in production of a short-lived mRNA or polypeptide, since we found that the UC620 mutation was phenocopied by complete deletion of the *MMA1* gene. ∆*mma1* cells, like UC620, showed defects in biochemical and morphological maturation of mucocysts, which were conditional with respect to growth conditions. Moreover, the defects in UC620 were fully rescued by expression of a wild-type copy of *MMA1* tagged with CFP.

Judging by transcript abundance, *MMA1* is expressed at a very low level. Consistent with this, we could not detect Mma1p tagged with 3 × GFP, when expressed under its endogenous promoter, and had to induce overexpression to visualize the protein in live cells. Analysis of those cells suggested that Mma1p is a cytosolic protein that partially associates with membranes, which may be small vesicles. This assignment as a cytosolic protein is also consistent with *in silico* predictions, namely the apparent absence of an endoplasmic reticulum translocation signal sequence or of any likely transmembrane domains. The precise role of Mma1p is unknown. Mucocyst biogenesis depends in part on endolysosomal trafficking, as judged by the requirement for a receptor in the VPS10 family, and several other proteins associated with trafficking to lysosome-related organelles (Briguglio *et al.* 2013; and our unpublished data). The small, relatively homogeneous, Mma1p-GFP-labeled puncta may therefore represent endosomes involved in mucocyst formation.

We have previously noted that the genes encoding all known luminal proteins in mucocysts were coexpressed together with a receptor responsible for their delivery and the processing enzymes responsible for their maturation (Briguglio *et al.* 2013; Kumar *et al.* 2014). Since the expression profile of *MMA1* closely matches that of the *SOR4* receptor, it appears that the phenomenon of coregulation extends to genes encoding cytosolic machinery in this pathway.

Although a mechanistic understanding of Mma1p action during mucocyst maturation is not yet in hand, our results demonstrate the power of forward genetics in *T. thermophila* to identify lineage-restricted genes that play essential roles in this pathway. It is particularly interesting that *MMA1* has no identifiable homolog in *P. tetraurelia*, another Oligohymenophorean ciliate that makes secretory granules called trichocysts. Trichocysts, like mucocysts, undergo biochemical and morphological maturation. Significantly, for virtually all known components of *Paramecium* trichocysts and *Tetrahymena* mucocysts, one can readily identify homologous (and likely orthologous) genes in the other organism. This is true for genes encoding the luminal cargo, processing enzymes, and membrane proteins involved in docking. Thus, the absence of an *MMA1* homolog in *Paramecium* is exceptional, and suggests the recent emergence of a novel mechanism in a largely conserved pathway within Oligohymenophorean ciliates.

## Supplementary Material

Supplemental Material
