# Supplementary material for: Whole Genome Sequencing Identifies a Novel Factor Required for Secretory Granule Maturation in Tetrahymena thermophila
Source: G3 (Bethesda). 2016 Jun 9;6(8):2505–16. doi: 10.1534/g3.116.028878 (PMC4978903; doi:10.1534/g3.116.028878)
Supplement: Supplemental Material [file supp_6_8_2505__index.html]

Whole Genome Sequencing Identifies a Novel Factor Required for Secretory Granule Maturation in Tetrahymena thermophila — Whole Genome Sequencing Identifies a Novel Factor Required for Secretory Granule Maturation in Tetrahymena thermophila — Supplemental Material 

# Whole Genome Sequencing Identifies a Novel Factor Required for Secretory Granule Maturation in *Tetrahymena thermophila*

## Supplemental Material for Kontur *et al.*, 2016

**Files in this Data Supplement:**

- Figure S1 - Cartoon of MMA1 disruption vector. (.jpg, 171 KB)
- Figure S2 - equences of primers used in this work. (.pptx, 297 KB)
- Figure S3 - Flowchart of genetic crosses to produce strains for sequencing. (.pptx, 51 KB)
- Figure S4 - Primary sequence alignment of *MMA1* homologs of four *Tetrahymena* species (*T. thermophila*, *T. malaccensis*, *T. ellioti*, and *T. borealis*). The GenBankTM Accession Numbers for *T. thermophila*, *T. malaccensis*, *T. ellioti*, and *T. borealis* GM are TTHERM\_00566910, EIA\_15322.1, EI7\_00003.1 and EI9\_14649.1, respectively. (.jpg, 571 KB)
