# Supplementary figures and images for: Whole Genome Sequencing Identifies a Novel Factor Required for Secretory Granule Maturation in Tetrahymena thermophila
Source: G3 (Bethesda). 2016 Jun 9;6(8):2505–16. doi: 10.1534/g3.116.028878 (PMC4978903; doi:10.1534/g3.116.028878)

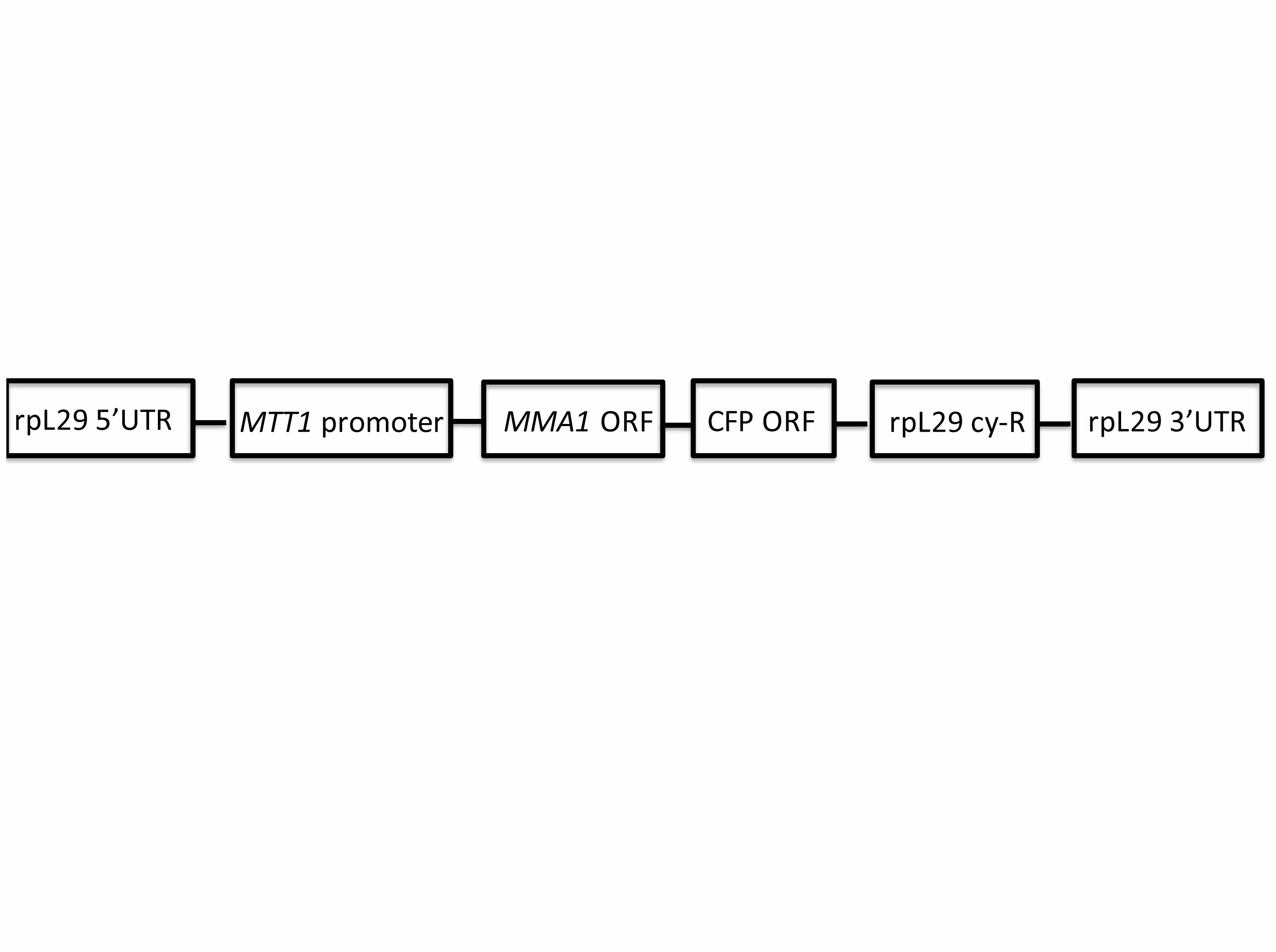

Supplement: Supplemental Material [file supp_g3.116.028878_FigureS1.jpg]

## Slide 1
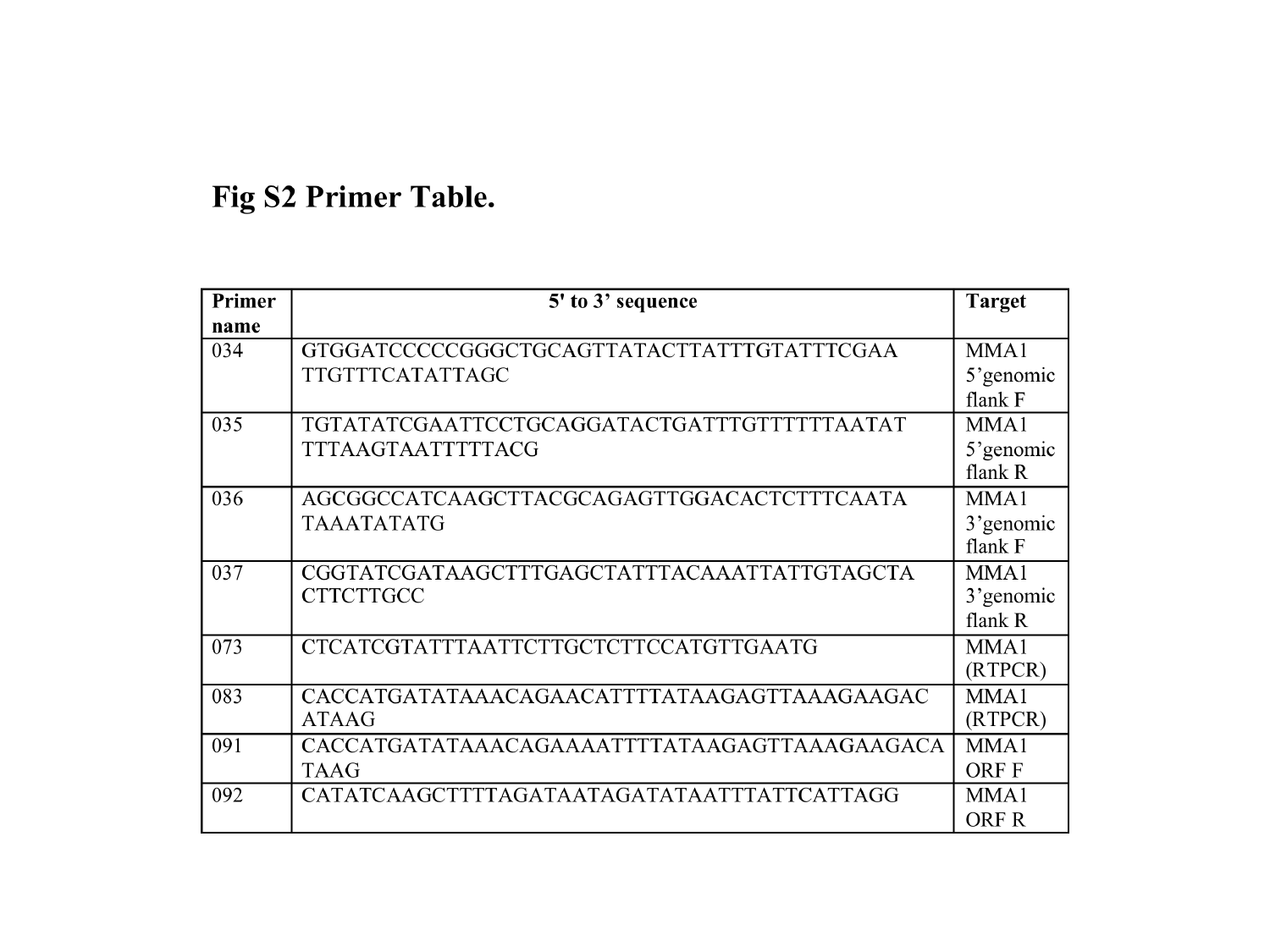

Supplement: Supplemental Material [file supp_g3.116.028878_FigureS2.pptx]

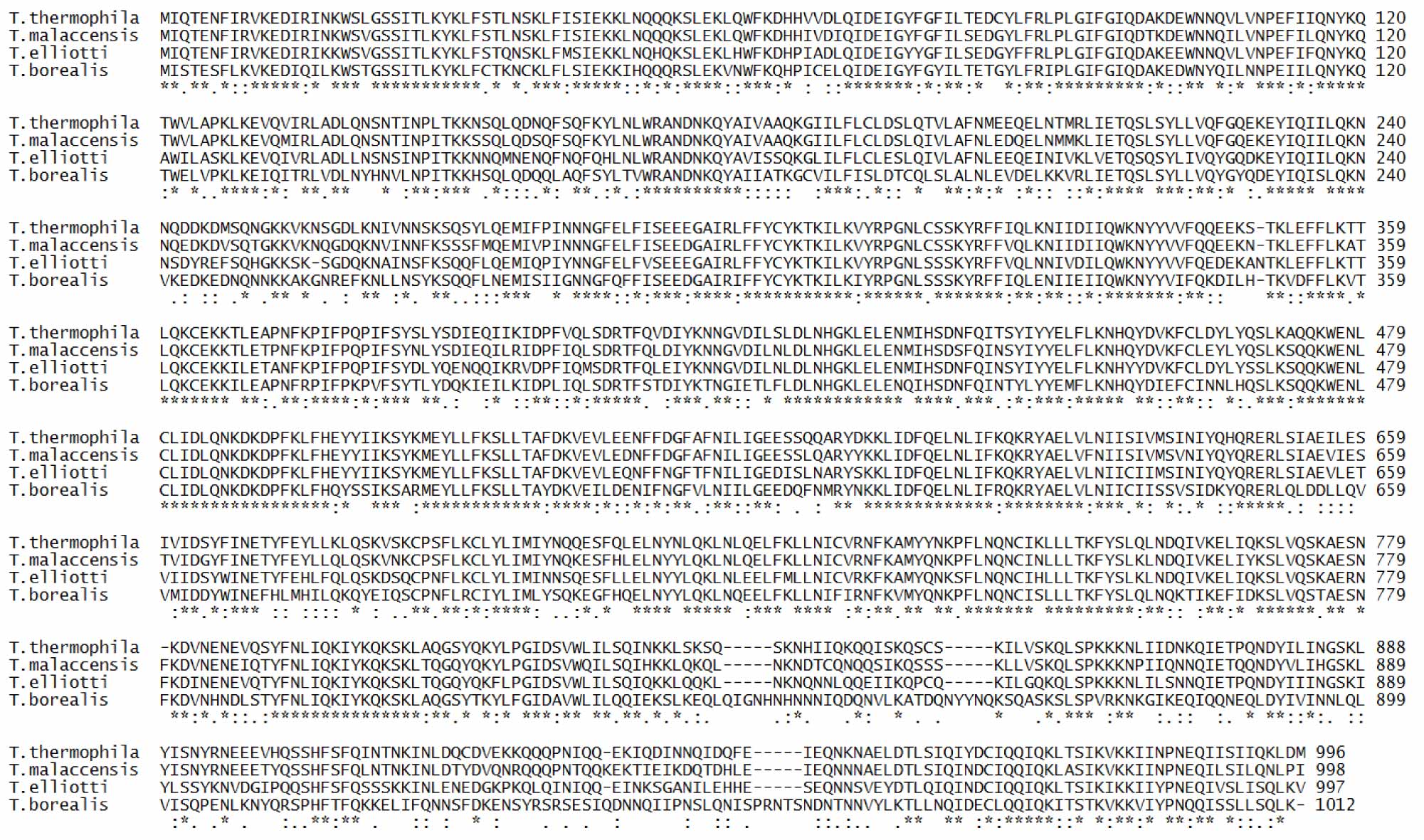

Supplement: Supplemental Material [file supp_g3.116.028878_FigureS4.jpg]
