# Supplementary material for: Whole Genome Sequencing Identifies a Novel Factor Required for Secretory Granule Maturation in Tetrahymena thermophila
Source: G3 (Bethesda). 2016 Jun 9;6(8):2505–16. doi: 10.1534/g3.116.028878 (PMC4978903; doi:10.1534/g3.116.028878)
Supplement: Supplemental Material [file supp_g3.116.028878_FigureS3.pptx]

## Slide 1
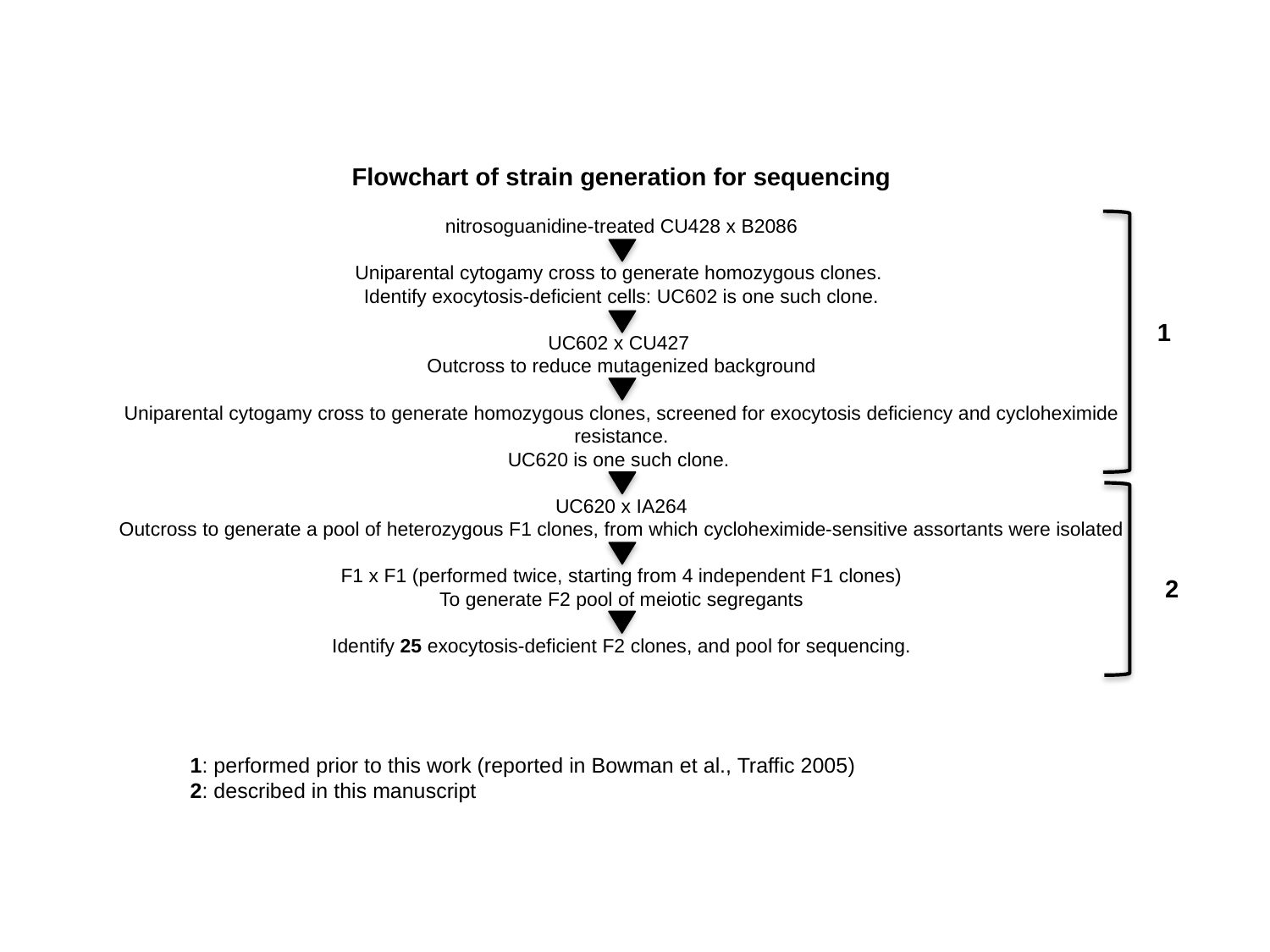

# Flowchart of strain generation for sequencing nitrosoguanidine-treated CU428 x B2086 Uniparental cytogamy cross to generate homozygous clones. Identify exocytosis-deficient cells: UC602 is one such clone. UC602 x CU427 Outcross to reduce mutagenized background Uniparental cytogamy cross to generate homozygous clones, screened for exocytosis deficiency and cycloheximide resistance. UC620 is one such clone. UC620 x IA264 Outcross to generate a pool of heterozygous F1 clones, from which cycloheximide-sensitive assortants were isolated F1 x F1 (performed twice, starting from 4 independent F1 clones) To generate F2 pool of meiotic segregants Identify 25 exocytosis-deficient F2 clones, and pool for sequencing.
1
2
1: performed prior to this work (reported in Bowman et al., Traffic 2005)
2: described in this manuscript
